# NY-ESO-1 as a potential immunotherapeutic target in renal cell carcinoma

**DOI:** 10.18632/oncotarget.2101

**Published:** 2014-06-13

**Authors:** Eva Giesen, Lucia B. Jilaveanu, Fabio Parisi, Yuval Kluger, Robert L. Camp, Harriet M. Kluger

**Affiliations:** ^1^ Yale Cancer Center, Yale University School of Medicine, New Haven, CT, U.S; ^2^ Department of Pathology, Yale University School of Medicine, New Haven, CT, U.S

**Keywords:** Cancer Testis Antigens, drug targets, kidney cancer, clear cell carcinoma, adoptive cell therapy

## Abstract

Background: Novel immune therapies targeting tumor specific antigens are being developed. Our purpose was to determine expression of the cancer testes antigen NY-ESO-1 in renal cell carcinoma (RCC), as NY-ESO-1 targeting approaches, particularly adoptive cell therapy, have not been evaluated in this disease.

Methods: We employed tissue microarrays containing >300 unique RCC cases and adjacent benign renal tissue to determine NY-ESO-1 expression using a quantitative immunofluorescence method. In addition, we studied NY-ESO-1 expression in 35 matched primary and metastatic RCC specimens to assess concordance between different tumor sites.

Results: NY-ESO-1 was highly expressed in a subset of RCCs. Expression in primary RCC specimens was significantly higher than adjacent normal renal tissue (P<0.0001) and higher in clear cell carcinomas than papillary RCC (P<0.0001). Expression levels in metastatic specimens were higher than in matched primary samples (P=0.0018), and the correlation between the two sites was modest (χ^2^=3.5, p=0.06).

Conclusions: Aberrant NY-ESO-1 expression seen in clear cell RCC suggests that NY-ESO-1 targeting approaches should be studied in this disease. Expression is higher in metastatic sites, and discordance between primary and metastatic sites in some patients suggests that patient selection for these therapies should be based on expression in metastatic rather than nephrectomy specimens.

## INTRODUCTION

Renal cell carcinoma (RCC) is a fairly common malignancy with a rising incidence, estimated at 64,770 new cases in the United States in 2012[1]. Despite increased incidental early detection, approximately 25% of patients develop metastatic disease (mRCC) [2]. RCC is felt to be one of the more immunoresponsive cancers, as a small percentage of mRCC tumors respond to cytokine therapies, such as interleukin-2 and interferon [3-6]. Despite recent advances in use of drugs that target vascular endothelial growth factor, its receptor and one of its downstream signaling mediators, mTOR, responses to these drugs remain of limited duration, while responses to high dose interleukin-2 tend to be durable [7-12]. Response rates to high-dose interleukin-2, however, are only in the order of 15-20%, and toxicities preclude its use in patients with co-morbidities, and there is therefore a great need for additional immune therapies that might have activity in this disease and carry the promise of durable responses. For example, recent use of inhibitors of the immune checkpoint inhibitor PD-1 in mRCC has resulted in durable responses in up to 30% of patients [13]. Additional immune-stimulatory approaches are also being tested in mRCC.

A number of newer immune therapies are being developed in other malignancies that target cancer/testis antigens (CTAs). CTAs can be expressed in malignant cells, whereas physiological expression is absent in normal tissue with the exception of the testis [14]. Targeting CTAs aberrantly expressed in malignant cells can provide a means for delivering selective anti-neoplastic therapy that is less toxic to normal cells, as suggested by Blagosklonny [15]. Although the biological functions of CTAs are mostly unknown, the expression pattern of CTAs makes them useful targets for specific immunotherapy. CTAs have been shown to have the ability to induce immune responses, one of the earlier examples being MAGE-1, which can be harnessed to activate T-cells in melanoma [16]. Other studies include cancer vaccines using other CTAs, such as Melan A and gp100, which have been used in clinical trials, resulting in immune responses, although with limited anti-tumor effect [17-18]. To date, over 50 cancer testis antigens have been identified, including NY-ESO-1, MAGE-1, BAGE, and GAGE. A number of immune therapies have recently been developed to target NY-ESO-1, including adoptive cell therapies and cell vaccines [19-21]. NY-ESO-1 has been harnessed for development of anti-cancer vaccines, primarily for melanoma, which is the disease that is believed to primarily express CTAs. Little is known, however, about expression of NY-ESO-1 in RCC.

NY-ESO-1 is a 22 kD hydrophobic protein that was discovered using SEREX (serological analysis of tumor antigens by recombinant expression cloning) analysis of an esophageal carcinoma [22]. The function of NY-ESO-1 is still unknown, but expression by cancer cells might reflect malignant properties, such as immortality, self-renewal, migratory ability and capacity to invade [23]. Spontaneous immune responses to NY-ESO-1, both cellular and humoral responses, is often detected in advanced cancer indicating that this molecule is immunogenic [24-26]. The frequency of anti-NY-ESO-1 antibody responses in patients with advanced NY-ESO-1-expressing tumors (including melanoma, lung cancer, bladder cancer, ovarian cancer, and breast cancer) is in the range of 25–50% [27-28]. Furthermore, antibody titers increase with disease progression and decrease upon removal of the tumor or tumor regression, although in breast cancer it is also expressed in ductal carcinoma in-situ [29-30]. The majority of patients, mostly with metastatic melanoma, that have an anti-NY-ESO-1 antibody response to their tumors have NY-ESO-1-speciﬁc CD8+ T-cells, as opposed to NY-ESO-1 antibody negative patients, who rarely develop CD8+ T cell responses against NY-ESO-1 [26-27, 31]. These findings reflect the immunogenicity of NY-ESO-1, making it an attractive target for cancer vaccines.

The prevalence of NY-ESO-1 expression is highly variable in different tumor types [14, 32]. Expression patterns, however, have not been extensively studied in RCC. Small cohort studies have been conducted using immunohistochemistry (IHC). One study, which included ten RCCs, reported the absence of NY-ESO-1 expression as analyzed by IHC [14]. Another study showed that NY-ESO-1 was expressed in 6 of 18 chromophobe RCCs and 15 of 17 oncocytomas [33]. Conflicting results were obtained by Bolli et. al who found NY-ESO-1 expression in only one out of 48 clear cell carcinomas, one out of 37 papillary RCCs and none of the 13 chromophobe RCCs, also using standard IHC [34]. Given the conflicting results in these small cohort RCC studies, we conducted a more comprehensive study of expression of NY-ESO-1 in a large cohort of over 300 RCC tumors of different histological subtypes with associated matched normal renal tissue. We also evaluated variability in expression in paired primary and metastatic tumors, as well as heterogeneity within these tumors. To enable us to conduct the latter studies we used a method of Automated QUantitative Analysis (AQUA) of immunofluorescence staining, which provides more objective and quantitative measure of NY-ESO-1 expression than standard IHC.

## RESULTS

### NY-ESO-1 staining patterns

**Figure 1:**
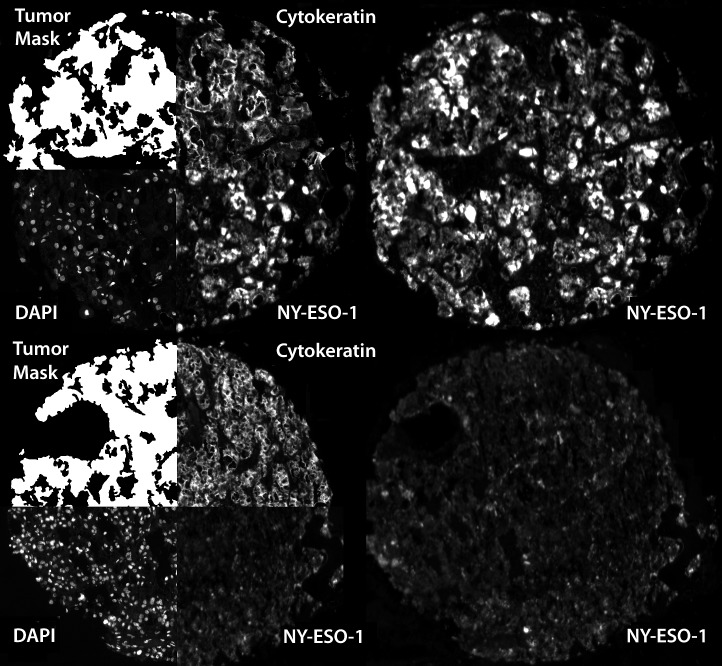
Automated, quantitative analysis (AQUA) of NY-ESO-1 Automated, quantitative analysis (AQUA) of histospots from high NY-ESO-1 expressing (AQUA score: 46.5) (upper-right) and low NY-ESO-1 expressing (AQUA score: 19.9) (lower-right) RCCs. Anti-cytokeratin conjugated to Cy2 was used to identify the cytoplasm and to define the tumor mask. DAPI was used to define the nuclear compartment and Cy5 was used to visualize the target (NY-ESO-1).

NY-ESO-1 expression, as measured by AQUA, was both cytoplasmic and nuclear. Figure 1 shows examples of strong and weak immunoreactivity of NY-ESO-1 in 2 histospots.

### NY-ESO-1 expression in a large cohort of nephrectomy specimens (Cohort A)

**Figure 2:**
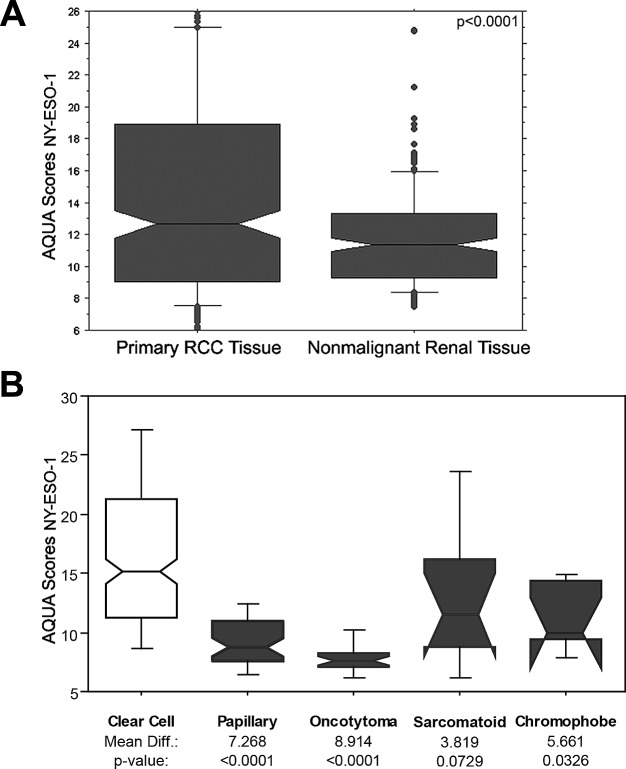
NY-ESO-1 expression in primary and nonmalignant renal specimens and in different histologic subtypes (A) Box plots representing the AQUA scores for NY-ESO-1 expression levels in primary RCC tumor and nonmalignant renal tissue. The horizontal bars correspond to the 5^th^, 50^th^ and 95^th^ percentiles. Primary RCC tumors has a significant higher NY-ESO-1 expression levels than corresponding nonmalignant renal tissue (p<0.0001). (B) Box plots representing AQUA scores in the various histologic subtypes showing higher expression in clear cell carcinomas than papillary RCCs, chromophobe tumors and oncocytomas.

This cohort included 334 primary RCC specimens of which 294 had adjacent matched normal renal tissues. Two sets of slides, each containing one core for each patient were stained, and scores were averaged. A high degree of core to core correlation was found between our two slides (R = 0.75). AQUA scores ranged from 5.046 to 46.463, with a mean of 14.876 for tumor tissues, and from 6.026 to 38.816 for nonmalignant tissues, with a mean of 11.934. Figure 2A shows a box plot comparing expression in nonmalignant and RCC tissue. Expression was significantly higher in malignant tumors than the adjacent normal kidney tissues (*t* statistic= 4.058, p= <0.0001). Expression differed among the histologic subtypes, as shown in Figure 2B; expression was significantly higher in clear cell carcinomas than papillary RCCs, choromophobe tumors and oncocytomas.

AQUA provides continuous output data, whereas clinical decisions are typically based on binarized information. We therefore defined “low” or “normal” AQUA scores as those that fall at or below the 95^th^ percentile score for normal renal tissue (an AQUA score of 16). Using this cutpoint, 42.5% of the clear cell, 18% of the sarcomatoid and 7% of the papillary RCCs had high NY-ESO-1 expression.

Using ANOVA we compared NY-ESO-1 expression and commonly used clinical/pathological variables. Expression was higher in low Fuhrman grade tumors than high Fuhrman grade tumors (grades 1 and 2 compared to 3 and 4 (p=0.028)). There was no significant association with stage III/IV disease (p=0.3071). No association was found between NY-ESO-1 expression and age at diagnosis (>50 versus younger than 50) (p=0.0873) or gender (p=0.3388). By Cox proportional hazards method there was no statistically significant association between NY-ESO-1 expression and overall survival.

### NY-ESO-1 expression in matched primary and metastatic samples (Cohort B)

**Figure 3:**
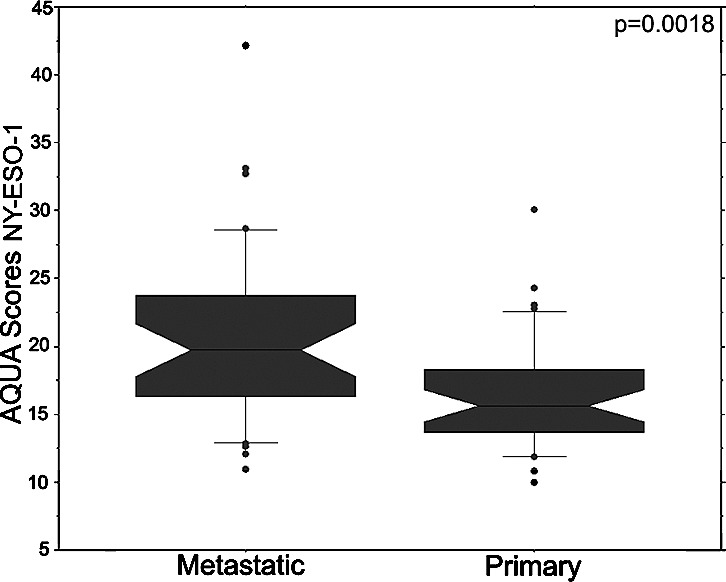
NY-ESO-1 expression in matched primary and metastatic specimens Box plots show NY-ESO-1 expression in matched primary and metastatic specimens from the same patients. Metastatic specimens have significantly higher NY-ESO-1 expression than the primary specimens (p=0.0018).

This cohort contains four cores from 35 matched primary and metastatic specimens (8 cores in total per patient) and was utilized to study differences in NY-ESO-1 expression in primary and metastatic specimens and NY-ESO-1 intra-tumor heterogeneity. AQUA scores ranged from 6.393 to 40.032 with a mean of 16.137 for primary RCC tissue, and from 6.398 to 47.367 for metastatic tissue, with a mean of 20.143. The higher expression levels in Cohort B likely reflects the fact that these were predominantly clear cell RCCs. NY-ESO-1 expression tended to be higher in metastatic RCC tissue than primary tumors (p=0.0018), as shown in the plot in Figure 3.

**Figure 4:**
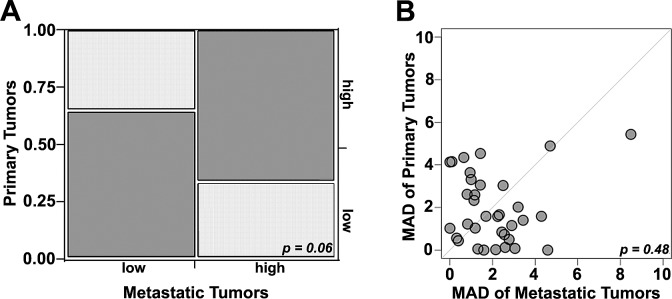
Estimates of NY-ESO-1 heterogeneity A)Chi-Sqare analysis comparing high and low NY-ESO-1 levels in matched primary and metastatic samples: Concordance (dark grey) was seen in 66% of cases while discordance (light grey) was seen in 34% (χ^2^ = 3.5, p=0.06). B) Comparison between heterogeneity within primary and metastatic specimens was estimated using a composite median absolute deviation (MAD). Each patient is represented by a dot. Dots above the diagonal represent patients with larger heterogeneity in the primary tumors, while dots below the diagonal represent greater heterogeneity in the corresponding metastatic tumors. The central diagonal grey line represents identical heterogeneity in primary and metastatic tumors. The Wilcoxon paired, two-sided signed rank test shows no significant difference between the heterogeneities of the primary and matched metastatic tumors (p = 0.48).

Seeing that RCC patients often have a nephrectomy prior to or after developing metastatic disease, we determined whether expression in primary specimens correlated with that of metastatic specimens. Conducting χ^2^ analysis of scores binarized by the median, we found modest concordance between the averaged scores from the matching primary and metastatic sites (χ^2^ = 3.5, p=0.06). Specifically, scores were high in both primary and metastatic sites in 12 of 35 cases (34%), low in both in 11 (31%) cases and discordant in 12 (34%). This indicates that NY-ESO-1 expression levels in primary tissue cannot reliably be used as a surrogate for determining expression levels in metastatic tissue and vice versa (Figure 4A).

Finally, to determine whether a biopsy specimen can be used to reliably measure NY-ESO-1 expression in a tumor, we studied the intra-tumor heterogeneity utilizing four different expression measurements for each tumor block. A composite median absolute deviation (MAD) was generated for each tumor. The MAD was variable across tumors (Figure 4B), indicating wide variability in the degree of heterogeneity. The Wilcoxon paired, two-sided signed rank test, showed no significant difference between the heterogeneities of the primary and matched metastatic tumors (p=0.48).

## DISCUSSION

In this study, we used two patient cohorts to determine expression of NY-ESO-1 in primary and metastatic samples and samples with variable histology. We used a quantitative method of immunofluorescence, which enables us to better determine heterogeneity of expression. Expression of NY-ESO-1 in primary RCC specimens is significantly higher than in normal renal tissue, and higher in clear cell carcinoma than other histologic subtypes. Sarcomatoid RCCs, which are now considered poorly differentiated clear cell RCCs also had high NY-ESO-1 expression. These findings suggest that NY-ESO-1 targeting immune therapies should be evaluated in clear cell carcinoma.

In our second cohort of matched primary and metastatic specimens, NY-ESO-1 expression was higher in metastatic specimens, supporting a potential role for NY-ESO-1 targeting immune therapies in patients with metastatic RCC. However, the modest correlation between expression in these matched specimens indicates that patient selection for NY-ESO-1 targeting immunotherapies should be based on expression in metastatic rather than primary tumor samples, if metastatic disease is primarily being targeted.

There was significant intra-tumor heterogeneity in both primary and metastatic samples. Although core needle biopsies are often done to diagnose metastatic disease, our findings indicate that a single core needle might be insufficient to establish presence or absence of NY-ESO-1 in metastatic RCC, and whole tumor specimens or additional cores might be needed. Studies of tumor heterogeneity in primary RCC using high throughput sequencing have suggested that the large, bulky primary tumors are highly heterogeneous [35]. Our study suggests that for NY-ESO-1 expression the degree of heterogeneity does not differ in primary and metastatic samples. This finding supports prior studies of other therapeutic targets in this cohort in which we found no difference in the degree of heterogeneity in primary and metastatic specimens [36].

The elevated expression of NY-ESO-1 in RCCs compared to normal renal tissue and the higher expression levels in metastatic specimens has important therapeutic implications. Despite recent advances in targeted therapies for patients with metastatic RCC, most patients will eventually succumb to their metastatic disease, and duration of response is typically in the order of months [7-12, 37-38]. Although new therapeutic approaches, such as targeting oxidative stress and intra-cellular metabolic pathways, are currently being studied, additional strategies of targeting the tumor microenvironment and activating immune cells are needed [39-40]. High dose interleukin-2 and inhibitors of PD-1 have resulted in durable responses, supporting a role for immune therapies for this disease.

Harnessing CTAs for vaccine or adoptive cell therapy has been done for other diseases, particularly melanoma, although CTAs are expressed in other disease as well, such as glioblastomas, colon, lung, liver, prostate cancer and other tumor types compared to non-malignant somatic cells [41-42]. CTA expression is necessary for these approaches, and our finding of high NY-ESO-1 expression in over 40% of clear cell RCCs suggests that these modalities should be evaluated in NY-ESO-1 expressing RCC. Clinical trials utilizing vaccines with NY-ESO-1 peptides, sometimes in combination with other therapies, are currently ongoing, and patient selection for these trials is frequently based on NY-ESO-1 expression. For example, a recent vaccine trial using recombinant vaccinia and fowlpox vectors expressing NY-ESO-1 showed that 72% of patients with unresectable melanoma whose tumors expressed NY-ESO-1 had clinical benefit from this therapy (defined as complete, partial, mixed response or disease stabilization) [21]. A trial of a human monoclonal antibody specific for the dendritic cell surface molecule DEC-205, fused to full-length NY-ESO-1, was recently completed (NCT00948961, www.clinicaltrials.gov). This trial was based on preclinical studies showing that this vaccine can induce CD8 and CD4 T cell responses [20]. Besides vaccination strategies, adoptive cell therapy with autologous T cells transduced with a T-cell receptor directed against NY-ESO-1 showed responses in previously treated patients with NY-ESO-1 bearing tumors; 4 out of 6 patients with advanced synovial cell sarcoma and 5 out of 11 patients with metastatic melanoma had an objective response, and this approach is now being studied in additional patients, using NY-ESO-1 tumor expression as a criterion for eligibility [19, 43].

In summary, given the new approaches to NY-ESO-1 targeting to treat a variety of malignancies, we studied expression patterns of NY-ESO-1 in primary and metastatic RCC. To the best of our knowledge, this is the first study to assess the expression of NY-ESO-1 in a large cohort of RCC specimens of variable histologic subtypes using a quantitative method. Elevated expression (above the 95^th^ expression level for normal kidney) was found in over 40% of clear cell carcinomas (>40%) and expression was higher in metastatic than primary specimens, supporting a role for NY-ESO-1 targeting in a subset of metastatic clear cell RCC patients. NY-ESO-1 expression is heterogeneous in both primary and metastatic samples, and correlation between corresponding primary and metastatic specimens was modest, indicating that eligibility for NY-ESO-1 targeting strategies should be determined based on expression in more than one core obtained from metastatic sites.

## MATERIALS AND METHODS

### Tissue microarray (TMA) construction and patient selection

Tumor tissues were collected from the Yale University Department of Pathology Archives. Specimens and clinical information were collected with the approval of a Yale University Institutional Review Board. TMAs were constructed using standard methods with cores each measuring 0.6 mm, spaced 0.8 mm apart. Two non-overlapping cohorts were used for this study, both previously described [36, 44]. Cohort A contains RCC cores from 330 patients, 294 with matching adjacent normal renal tissue from nephrectomies carried out between 1987 and 1999. Tumors were represented by 2 cores from different areas of the specimen and placed in two TMA blocks. Histologic subtypes included clear cell (71%), papillary (14%), chromophobe (2%), mixed histology (4%), oncocytomas (6%), and sarcomatoid tumors (3%). Fifty-six percent (56%) had stage I disease, 8% stage II, 8% stage III and 28% had stage IV disease. Fuhrman nuclear grade I was seen in 12%, 52% had grade II, 27% grade III and 9% grade IV. Age at diagnosis was 25 to 87 years (median, 63). Information on calcium, LDH, Hb, and performance status was not available for this cohort of patients. None of these patients was treated with drugs targeting the VEGF or mTOR pathways.

Cohort B consists of matched primary and metastatic RCC tumors of thirty-five patients, who had undergone resections between 1972 and 2011. Each tumor site was represented by four cores from different areas of the specimen (8 cores of each patient in total). Histological subtypes included clear cell (91.2%) and mixed papillary and clear cell histology (two cases, 8.8%).

### Immunofluorescent staining

One set of slides per cohort was stained for Automated QUantitative Analysis (AQUA) for NY-ESO-1 expression, as described previously [45-46]. In brief, slides were incubated overnight with mouse monoclonal anti-human CTAG1B/NY-ESO-1 (Novus Biologicals, Littleton, CO, dilution 1:200). Antibody specificity was determined by immunoblot of a panel of melanoma cell lines with variable NY-ESO-1 expression (not shown). Goat anti-mouse horseradish peroxidase-decorated polymer backbone (Envision, Dako, Carpinteria, CA) was used as a secondary reagent. For NY-ESO-1 visualization, slides were incubated with Cyanine5-tyramide (Perkin Elmer, Waltham, MA) in the supplied amplification buffer. A tumor mask was created by simultaneous incubation with a cocktail containing rabbit anti-cytokeratin (Dako, Carpinteria, CA, dilution 1:100), anti-CAIX (Novus Biologicals, Littleton, CO, dilution 1:2000) and Streptavidin HRP (Sigma, St. Louis, MO, dilution 1:100) for 2 hours. Goat anti-rabbit HRP-decorated polymer backbone (Envision, Dako, Carpinteria, CA, USA) was used as a secondary reagent. Cyanine2-tyramide (Perkin Elmer, Waltham, MA, USA) was used to visualize tumor mask. The nuclear compartment was visualized with 4, 6-diamidine-2-phenylindole (DAPI) (Invitrogen, Carlsbad, CA, dilution 1:500). Coverslips were mounted with ProLong Gold anti-fade reagent with DAPI (Invitrogen, Carlsbad, CA).

### Image Acquisition and Analysis

Images were acquired and analyzed using algorithms that have been previously described [46]. Please see Fig.1. Monochromatic, high-resolution (1024 × 1024 pixel) images were obtained of each histospot. Tumor mask (and normal kidney tissue mask) was created from the Cy2 signal and DAPI was used to identify the nuclei. The target signal (NY-ESO-1) visualized by Cy5, was compartmentalized and expressed as the average signal intensity within the assayed component (AQUA score), with scores on a scale of 0–255.

### Statistical analysis

Histospots were excluded from the analysis if the tumor mask represented <3% of the histospot area, if there was anomalous staining (lacking Cy2 or DAPI signal) or abundant necrosis. For comparison with clinical variables, scores from replicate histospots were averaged.

Statview and JMP 9.0 software was used (SAS Institute, Cary, NC). Associations between continuous NY-ESO-1 values and pathological parameters were assessed using analysis of variance (ANOVA). Correlations between the AQUA scores of matched primary and metastatic histospots were calculated by the log rank method. Associations with survival were determined using the Cox proportional hazards method.

## References

[1] Siegel R, DeSantis C, Virgo K, Stein K, Mariotto A, Smith T, Cooper D, Gansler T, Lerro C, Fedewa S, Lin C, Leach C, Cannady RS, Cho H, Scoppa S, Hachey M (2012). Cancer treatment and survivorship statistics, 2012. CA: a cancer journal for clinicians.

[2] Gupta K, Miller JD, Li JZ, Russell MW, Charbonneau C (2008). Epidemiologic and socioeconomic burden of metastatic renal cell carcinoma (mRCC): a literature review. Cancer treatment reviews.

[3] Fisher RI, Rosenberg SA, Fyfe G (2000). Long-term survival update for high-dose recombinant interleukin-2 in patients with renal cell carcinoma. The cancer journal from Scientific American.

[4] Shablak A, Sikand K, Shanks JH, Thistlethwaite F, Spencer-Shaw A, Hawkins RE (2011). High-dose interleukin-2 can produce a high rate of response and durable remissions in appropriately selected patients with metastatic renal cancer. J Immunother.

[5] Klapper JA, Downey SG, Smith FO, Yang JC, Hughes MS, Kammula US, Sherry RM, Royal RE, Steinberg SM, Rosenberg S (2008). High-dose interleukin-2 for the treatment of metastatic renal cell carcinoma: a retrospective analysis of response and survival in patients treated in the surgery branch at the National Cancer Institute between 1986 and 2006. Cancer.

[6] McDermott DF, Regan MM, Clark JI, Flaherty LE, Weiss GR, Logan TF, Kirkwood JM, Gordon MS, Sosman JA, Ernstoff MS, Tretter CP, Urba WJ, Smith JW, Margolin KA, Mier JW, Gollob JA, et al (2005). Randomized phase III trial of high-dose interleukin-2 versus subcutaneous interleukin-2 and interferon in patients with metastatic renal cell carcinoma. Journal of clinical oncology: official journal of the American Society of Clinical Oncology.

[7] Motzer RJ, Hutson TE, Tomczak P, Michaelson MD, Bukowski RM, Rixe O, Oudard S, Negrier S, Szczylik C, Kim ST, Chen I, Bycott PW, Baum CM, Figlin RA (2007). Sunitinib versus interferon alfa in metastatic renal-cell carcinoma. The New England journal of medicine.

[8] Sternberg CN, Davis ID, Mardiak J, Szczylik C, Lee E, Wagstaff J, Barrios CH, Salman P, Gladkov OA, Kavina A, Zarba JJ, Chen M, McCann L, Pandite L, Roychowdhury DF, Hawkins RE (2010). Pazopanib in locally advanced or metastatic renal cell carcinoma: results of a randomized phase III trial. Journal of clinical oncology: official journal of the American Society of Clinical Oncology.

[9] Rini BI, Escudier B, Tomczak P, Kaprin A, Szczylik C, Hutson TE, Michaelson MD, Gorbunova VA, Gore ME, Rusakov IG, Negrier S, Ou YC, Castellano D, Lim HY, Uemura H, Tarazi J (2011). Comparative effectiveness of axitinib versus sorafenib in advanced renal cell carcinoma (AXIS): a randomised phase 3 trial. Lancet.

[10] Rini BI, Halabi S, Rosenberg JE, Stadler WM, Vaena DA, Ou SS, Archer L, Atkins JN, Picus J, Czaykowski P, Dutcher J, Small EJ (2008). Bevacizumab plus interferon alfa compared with interferon alfa monotherapy in patients with metastatic renal cell carcinoma: CALGB 90206. Journal of clinical oncology: official journal of the American Society of Clinical Oncology.

[11] Motzer RJ, Escudier B, Oudard S, Hutson TE, Porta C, Bracarda S, Grunwald V, Thompson JA, Figlin RA, Hollaender N, Urbanowitz G, Berg WJ, Kay A, Lebwohl D, Ravaud A (2008). Efficacy of everolimus in advanced renal cell carcinoma: a double-blind, randomised, placebo-controlled phase III trial. Lancet.

[12] Hudes G, Carducci M, Tomczak P, Dutcher J, Figlin R, Kapoor A, Staroslawska E, Sosman J, McDermott D, Bodrogi I, Kovacevic Z, Lesovoy V, Schmidt-Wolf IG, Barbarash O, Gokmen E, O'Toole T (2007). Temsirolimus, interferon alfa, or both for advanced renal-cell carcinoma. The New England journal of medicine.

[13] Topalian SL, Hodi FS, Brahmer JR, Gettinger SN, Smith DC, McDermott DF, Powderly JD, Carvajal RD, Sosman JA, Atkins MB, Leming PD, Spigel DR, Antonia SJ, Horn L, Drake CG, Pardoll DM (2012). Safety, activity, and immune correlates of anti-PD-1 antibody in cancer. The New England journal of medicine.

[14] Jungbluth AA, Chen YT, Stockert E, Busam KJ, Kolb D, Iversen K, Coplan K, Williamson B, Altorki N, Old LJ (2001). Immunohistochemical analysis of NY-ESO-1 antigen expression in normal and malignant human tissues. Int J Cancer.

[15] Blagosklonny MV (2003). Tissue-selective therapy of cancer. Br J Cancer.

[16] van der, Bruggen P, Traversari C, Chomez P, Lurquin C, De Plaen E, Van den, Eynde B, Knuth A, Boon T (1991). A gene encoding an antigen recognized by cytolytic T lymphocytes on a human melanoma. Science.

[17] Jager E, Karbach J, Gnjatic S, Neumann A, Bender A, Valmori D, Ayyoub M, Ritter E, Ritter G, Jager D, Panicali D, Hoffman E, Pan L, Oettgen H, Old LJ, Knuth A (2006). Recombinant/fowlpox NY-ESO-1 vaccines induce both humoral and cellular NY-ESO-1-specific immune responses in cancer patients. Proc Natl Acad Sci U S A.

[18] Steele JC, Rao A, Marsden JR, Armstrong CJ, Berhane S, Billingham LJ, Graham N, Roberts C, Ryan G, Uppal H, Walker C, Young LS, Steven NM (2011). Phase I/II trial of a dendritic cell vaccine transfected with DNA encoding melan A and gp100 for patients with metastatic melanoma. Gene Ther.

[19] Robbins PF, Morgan RA, Feldman SA, Yang JC, Sherry RM, Dudley ME, Wunderlich JR, Nahvi AV, Helman LJ, Mackall CL, Kammula US, Hughes MS, Restifo NP, Raffeld M, Lee CC, Levy CL (2011). Tumor regression in patients with metastatic synovial cell sarcoma and melanoma using genetically engineered lymphocytes reactive with NY-ESO-1. Journal of clinical oncology: official journal of the American Society of Clinical Oncology.

[20] Tsuji T, Matsuzaki J, Kelly MP, Ramakrishna V, Vitale L, He LZ, Keler T, Odunsi K, Old LJ, Ritter G, Gnjatic S (2011). Antibody-targeted NY-ESO-1 to mannose receptor or DEC-205 in vitro elicits dual human CD8+ and CD4+ T cell responses with broad antigen specificity. J Immunol.

[21] Odunsi K, Matsuzaki J, Karbach J, Neumann A, Mhawech-Fauceglia P, Miller A, Beck A, Morrison CD, Ritter G, Godoy H, Lele S, duPont N, Edwards R, Shrikant P, Old LJ, Gnjatic S (2012). Efficacy of vaccination with recombinant vaccinia and fowlpox vectors expressing NY-ESO-1 antigen in ovarian cancer and melanoma patients. Proc Natl Acad Sci U S A.

[22] Chen YT, Boyer AD, Viars CS, Tsang S, Old LJ, Arden KC (1997). Genomic cloning and localization of CTAG, a gene encoding an autoimmunogenic cancer-testis antigen NY-ESO-1, to human chromosome Xq28. Cytogenet Cell Genet.

[23] Nicholaou T, Ebert L, Davis ID, Robson N, Klein O, Maraskovsky E, Chen W, Cebon J (2006). Directions in the immune targeting of cancer: lessons learned from the cancer-testis Ag NY-ESO-1. Immunol Cell Biol.

[24] Jager E, Gnjatic S, Nagata Y, Stockert E, Jager D, Karbach J, Neumann A, Rieckenberg J, Chen YT, Ritter G, Hoffman E, Arand M, Old LJ, Knuth A (2000). Induction of primary NY-ESO-1 immunity: CD8+ T lymphocyte and antibody responses in peptide-vaccinated patients with NY-ESO-1+ cancers. Proc Natl Acad Sci U S A.

[25] Ebert LM, Liu YC, Clements CS, Robson NC, Jackson HM, Markby JL, Dimopoulos N, Tan BS, Luescher IF, Davis ID, Rossjohn J, Cebon J, Purcell AW, Chen W (2009). A long, naturally presented immunodominant epitope from NY-ESO-1 tumor antigen: implications for cancer vaccine design. Cancer Res.

[26] Jager E, Chen YT, Drijfhout JW, Karbach J, Ringhoffer M, Jager D, Arand M, Wada H, Noguchi Y, Stockert E, Old LJ, Knuth A (1998). Simultaneous humoral and cellular immune response against cancer-testis antigen NY-ESO-1: definition of human histocompatibility leukocyte antigen (HLA)-A2-binding peptide epitopes. J Exp Med.

[27] Caballero OL, Chen YT (2009). Cancer/testis (CT) antigens: potential targets for immunotherapy. Cancer science.

[28] Stockert E, Jager E, Chen YT, Scanlan MJ, Gout I, Karbach J, Arand M, Knuth A, Old LJ (1998). A survey of the humoral immune response of cancer patients to a panel of human tumor antigens. J Exp Med.

[29] Jager E, Stockert E, Zidianakis Z, Chen YT, Karbach J, Jager D, Arand M, Ritter G, Old LJ, Knuth A (1999). Humoral immune responses of cancer patients against “Cancer-Testis” antigen NY-ESO-1: correlation with clinical events. Int J Cancer.

[30] Caballero OL, Shousha S, Zhao Q, Simpson AJ, Coombes RC, Nevillev AM (2014). Expression of Cancer/Testis genes in ductal carcinoma in situ and benign lesions of the breast Oncoscience.

[31] Jager E, Nagata Y, Gnjatic S, Wada H, Stockert E, Karbach J, Dunbar PR, Lee SY, Jungbluth A, Jager D, Arand M, Ritter G, Cerundolo V, Dupont B, Chen YT, Old LJ (2000). Monitoring CD8 T cell responses to NY-ESO-1: correlation of humoral and cellular immune responses. Proc Natl Acad Sci U S A.

[32] Scanlan MJ, Simpson AJ, Old LJ (2004). The cancer/testis genes: review, standardization, and commentary. Cancer Immun.

[33] Demirovic A, Dzombeta T, Tomas D, Spajic B, Pavic I, Hudolin T, Milosevic M, Cupic H, Kruslin B (2010). Immunohistochemical expression of tumor antigens MAGE-A3/4 and NY-ESO-1 in renal oncocytoma and chromophobe renal cell carcinoma. Pathology, research and practice.

[34] Bolli M, Schultz-Thater E, Zajac P, Guller U, Feder C, Sanguedolce F, Carafa V, Terracciano L, Hudolin T, Spagnoli GC, Tornillo L (2005). NY-ESO-1/LAGE-1 coexpression with MAGE-A cancer/testis antigens: a tissue microarray study. Int J Cancer.

[35] Gerlinger M, Rowan AJ, Horswell S, Larkin J, Endesfelder D, Gronroos E, Martinez P, Matthews N, Stewart A, Tarpey P, Varela I, Phillimore B, Begum S, McDonald NQ, Butler A, Jones D (2012). Intratumor heterogeneity and branched evolution revealed by multiregion sequencing. The New England journal of medicine.

[36] Aziz SA, Sznol JA, Adeniran A, Parisi F, Kluger Y, Camp RL, Kluger HM (2013). Expression of drug targets in primary and matched metastatic renal cell carcinoma tumors. BMC Clin Pathol.

[37] Escudier B, Eisen T, Stadler WM, Szczylik C, Oudard S, Staehler M, Negrier S, Chevreau C, Desai AA, Rolland F, Demkow T, Hutson TE, Gore M, Anderson S, Hofilena G, Shan M, et al (2009). Sorafenib for treatment of renal cell carcinoma: Final efficacy and safety results of the phase III treatment approaches in renal cancer global evaluation trial. Journal of clinical oncology: official journal of the American Society of Clinical Oncology.

[38] Escudier B, Bellmunt J, Negrier S, Bajetta E, Melichar B, Bracarda S, Ravaud A, Golding S, Jethwa S, Sneller V (2010). Phase III trial of bevacizumab plus interferon alfa-2a in patients with metastatic renal cell carcinoma (AVOREN): final analysis of overall survival. Journal of clinical oncology: official journal of the American Society of Clinical Oncology.

[39] Raninga P, Di Trapani G, Tonissen KF (2014). Cross talk between two antioxidant systems, Thioredoxin and DJ-1: consequences for cancer Oncoscience.

[40] Zaravinos A, Pieri AM, Mourmouras N, Anastasiadou N, Zouvani I, Delakas D, Deltas C (2014). Altered metabolic pathways in clear cell renal cell carcinoma: A meta-analysis and validation study focused on the deregulated genes and their associated networks. Oncoscience.

[41] Freitas M, Malheiros S, Stavale JN, Biassi TP, Zamuner FT, de Souza, Begnami M, Soares FA, Vettore AL (2013). Expression of cancer/testis antigens is correlated with improved survival in glioblastoma. Oncotarget.

[42] Sammut SJ, Feichtinger J, Stuart N, Wakeman JA, Larcombe L, McFarlane RJ (2014). A novel cohort of cancer-testis biomarker genes revealed through meta-analysis of clinical data sets. Oncoscience.

[43] Hunder NN, Wallen H, Cao J, Hendricks DW, Reilly JZ, Rodmyre R, Jungbluth A, Gnjatic S, Thompson JA, Yee C (2008). Treatment of metastatic melanoma with autologous CD4+ T cells against NY-ESO-1. The New England journal of medicine.

[44] Jilaveanu LB, Sznol J, Aziz SA, Duchen D, Kluger HM, Camp RL (2012). CD70 expression patterns in renal cell carcinoma. Human pathology.

[45] McCarthy MM, DiVito KA, Sznol M, Kovacs D, Halaban R, Berger AJ, Flaherty KT, Camp RL, Lazova R, Rimm DL, Kluger HM (2006). Expression of tumor necrosis factor--related apoptosis-inducing ligand receptors 1 and 2 in melanoma. Clinical cancer research: an official journal of the American Association for Cancer Research.

[46] Camp RL, Chung GG, Rimm DL (2002). Automated subcellular localization and quantification of protein expression in tissue microarrays. Nature medicine.

